# Regulation and functions of mammalian LATS/NDR kinases: looking beyond canonical Hippo signalling

**DOI:** 10.1186/2045-3701-3-32

**Published:** 2013-08-28

**Authors:** Alexander Hergovich

**Affiliations:** 1 Tumour Suppressor Signalling Networks laboratory, UCL Cancer Institute, University College London, London WC1E 6BT, UK

**Keywords:** Mammalian Hippo signalling, LATS1/2, NDR1/2, STK38/STK38L, Phosphorylation, Regulatory mechanisms, Kinase substrates, Intracellular functions, T-loop, Hydrophobic motif

## Abstract

The metazoan Hippo pathway is an essential tumour suppressor signalling cascade that ensures normal tissue growth by co-ordinating cell proliferation, cell death and cell differentiation. Over the past years, various genetic and biochemical studies in *Drosophila* and mammals have defined a conserved core Hippo signalling module, composed of members of the Ste20-like kinase, the MOB co-activator and the AGC kinase families. In *Drosophila*, stimulated Hippo kinase phosphorylates and thereby activates the Mats/Warts complex, which consequently phosphorylates and inactivates the transcriptional co-activator Yorkie. In mammals, the counterparts of the Hippo/Mats/Warts/Yorkie cascade, namely MST1/2, MOB1A/B, LATS1/2 and YAP/TAZ, function in a similar fashion. These canonical Hippo pathways are so highly conserved that human MST2, hMOB1A and LATS1 can compensate for the loss of Hippo, Mats and Warts in flies. However, recent reports have shown that Hippo signalling is more diverse and complex, in particular in mammals. In this review, we summarize our current understanding of mammalian LATS1/2 kinases together with their closest relatives, the NDR1/2 kinases. The regulation of the LATS/NDR family of kinases will be discussed, followed by a summary of all currently known LATS/NDR substrates. Last, but not least, the biological roles of LATS/NDR kinases will be reviewed with specific emphasis on recent discoveries of canonical and non-canonical LATS/NDR functions in the extended Hippo pathway.

## Introduction

The Hippo tumour suppressor pathway regulates cell proliferation, cell death and cell differentiation in multicellular organisms to ensure normal tissue development [1,2]. The final output of Hippo signalling is the inhibition of the transcriptional co-activators Yorkie and YAP (yes-associated protein) / TAZ (transcriptional co-activator with PDZ-binding motif) in flies and mammals, respectively [3]. Essentially, the core Hippo signalling module is composed of members of the Ste20-like kinase, the MOB (mps one binder) co-activator and the AGC (protein kinase A(PKA)/PKG/PKC-like) kinase families [4]. In *Drosophila*, the stimulated Ste20-like Hippo kinase phosphorylates and thereby activates a complex composed of Mats (mob as tumour suppressor) and the AGC Warts kinase. The Mats/Warts complex then phosphorylates and inactivates the transcriptional co-activator Yorkie. In mammals, the counterparts of the Hippo/Mats/Warts/Yorkie cascade, namely MST1/2 (mammalian Ste20-like serine/threonine kinases 1/2), MOB1A/B, LATS1/2 (large tumour suppressor 1/2) and YAP/TAZ, function in a similar fashion. While all core Hippo components upstream of Yorkie have been defined as tumour suppressors in flies, Yorkie displays proto-oncogenic properties [5]. In mammals, genetic studies provided the same picture, namely that loss of MST1/2 [6], MOB1A/B [7], or LATS1 [8] results in tumour growth, while YAP overexpression is sufficient to induce tumours [9]. Therefore, mammalian Hippo signalling has been defined as a tumour suppressor pathway that is essential for the control of the proto-oncoproteins YAP/TAZ [10,11]. Since the functions and regulation of YAP/TAZ have been reviewed recently [3], we will focus in this review only on the LATS-mediated regulation of YAP/TAZ. Furthermore, for a discussion of the crosstalk between Hippo and Wnt/TGFβ signalling we refer the reader to a recent overview provided by Varelas and Wrana [12].

*Drosophila* Warts and mammalian LATS1/2 kinases are members of the serine/threonine AGC class of protein kinases [13]. More specifically, LATS1/2 have been classified as a subgroup of AGC kinases together with NDR1/2 (nuclear Dbf2 related kinases 1/2; also known as STK38/STK38L), based on two unique characteristics, a conserved N-terminal regulatory domain (NTR) and an insert between subdomains VII and VIII of the catalytic kinase domain [4]. Like other AGC kinases, LATS/NDR kinases are regulated by phosphorylation on the activation segment motif (AS; also referred to as T-loop) and a C-terminally located hydrophobic motif (HM), which will be discussed later in more detail. Initially, our understanding of LATS/NDR kinases was mainly based on genetic studies performed in yeast and flies [4]. Therefore, before focusing entirely on our current understanding of mammalian LATS/NDR kinases, we feel that it is appropriate to give a brief historic overview of key discoveries with respect to core Hippo signalling.

In budding and fission yeast, the LATS/NDR kinases Dbf2p and Sid2p were described as central members of MEN/SIN signalling which is essential for proper mitotic exit [14], while the LATS/NDR kinases Cbk1 and Orb6 were attributed functions in the regulation of morphogenesis [4]. In *Drosophila*, Warts and Tricornered (the counterparts of mammalian LATS1/2 and NDR1/2, respectively) were discovered more than 10 years ago [15-17], and subsequent studies established Warts and Trc (Tricornered) as key players in Hippo signalling and dendritic tiling, respectively [5,18]. Significantly, human LATS1 and NDR1 were able to rescue the loss of Warts and Trc function, respectively [19,20], strongly suggesting that LATS/NDR functions are conserved between flies and mammals [4]. In 2002, Tapon et al. reported the regulation of Warts by the scaffolding protein Salvador (also known as WW45 in mammals) [21]. However, the breakthrough for Hippo signalling were five publications in 2003 [22-26], demonstrating that the Ste20-like kinase Hippo is functioning upstream of Warts in tissue growth control. Significantly, one study even showed that human MST2 can compensate for Hippo loss of function [26]. Subsequently, Lai et al. described Mats (also known as dMOB1) as a key regulator of Warts downstream of Hippo [27]. The same study also showed that hMOB1A can rescue the lethality associated with loss of Mats function in flies [27]. Next, the Pan laboratory discovered first that proto-oncogenic Yorkie was functioning downstream of Hippo/Warts/Mats signalling [28], and then also showed the phospho-regulation of Yorkie by Warts [9]. These studies enabled then the Pan and Guan laboratories to establish how YAP (one of two human counterparts of Yorkie) was regulated by LATS1/2 phosphorylation in mammalian cells [9,29], which will be discussed in more detail later.

Since these discoveries were published nearly a decade ago, the *Drosophila* community has continued to discover positive and negative regulators of Hippo signalling, which has been reviewed in detail recently [5]. Based on our personal interest in kinase signalling in the Hippo pathway, we will only briefly mention how additional kinases influence Hippo signalling, besides Hippo/MST and Warts/LATS kinases. Recently, the kinases Tao (thousand and one) and HIPKs (homeodomain-interacting protein kinases) were shown to regulate Hippo activity [30,31] and Yki function [32,33], respectively. Both regulatory mechanisms appear to be conserved from flies to humans, since human TAO1 can also activate MST2 [31], and HIPK2 promotes YAP activity in human cells [33]. In addition, Sik (salt-inducible kinase) has recently been shown to be required for Hippo signalling by phosphorylating Salvador in flies [34]. However, while human SIK2 can also inhibit YAP activity in HEK293 cells, the molecular mechanism must be different between flies and mammals, since the phosphorylation site in *Drosophila* Salvador is not conserved in mammals [34]. This molecular difference was not so surprising, since the transcriptional outputs of Hippo signalling are known to differ significantly between flies and mammalian cells [35], and Bossuyt et al. recently reported fundamental differences in the upstream regulatory mechanisms of Hippo signalling between *Drosophila* and mammals [36]. Nevertheless, in spite of this growing complexity upstream of Hippo, *Drosophila* genetics still supports a linear Mats/Warts/Yorkie cascade downstream of Hippo [5]. In light of this canonical Hippo signalling (Hippo signals to Mats/Warts, which then regulates Yorkie), we review here the regulation and functions of mammalian LATS1/2 kinases.

### Regulation of mammalian LATS/NDR kinases

In spite of the fast progress with deciphering Warts and LATS1/2 functions in flies and mammals, the mechanism of NDR1/2 regulation by phosphorylation currently must serve as a model for LATS1/2 regulation [4,37]. Therefore, we will first describe how mammalian NDR1/2 kinases are regulated, before highlighting our limited understanding of the regulatory mechanism of mammalian LATS1/2 (see Table 1). As already mentioned, NDR1/2 kinases are members of a subgroup of AGC kinases containing two key regulatory phosphorylation sites [38], the Ser281/282 AS and Thr444/Thr442 HM, respectively [4]. Binding of hMOB1A/B (the human counterparts of Mats) to the NTR domain of NDR1/2, which is highly conserved from yeast to humans and located N-terminally of the catalytic domain [4,39], increases the auto-phosphorylation activity of NDR1/2, thereby elevating Ser281/282 phosphorylation on NDR1/2 [40]. In contrast, HM phosphorylation of NDR1/2 is performed independently of NDR1/2 kinase activity [41]. MST1/2 (the human counterparts of Hippo) and MST3, another member of the MST kinase family [42], can phosphorylate NDR1/2 on Thr444/442 [43-46]. These S281 and Thr444 phosphorylations occur independently of Insulin/IGF-1/PDK1 signalling [38], but are counteracted by PP2A (protein phosphatase type 2A), since recombinant PP2A dephosphorylates NDR1 *in vitro*[38] and treatment of cells with okadaic acid (OA), a potent PP2A inhibitor, increases NDR1/2 phosphorylation levels [38,41,47]. NDR1/2 versions carrying S281/282A, S281D, S281E, T444/442A, T444D or T444E mutations have dramatically reduced kinase activities upon OA treatment [38,47], suggesting that both regulatory sites on NDR1/2 are essential for NDR1/2 kinase activity, but cannot be mimicked by standard phospho-mimetic alterations. Taken together, these biochemical studies conducted by the Hemmings laboratory defined the following regulatory mechanisms: (1) binding of hMOB1A/B to the NTR of NDR1/2 triggers auto-phosphorylation of NDR1/2 on Ser281/282, (2) MST1/2/3 kinases phosphorylate NDR1/2 on Thr444/442, and (3) PP2A can dephosphorylate both sites on NDR1/2 .

**Table 1:** Overview of the regulation of mammalian LATS/NDR kinases by phosphorylation

**Kinase(s)**	**Upstream kinase**	**Site(s)**	**Role of phosphorylation**
NDR1/2	Auto-phosphorylation	Ser281/Ser282	Essential for kinase activity [38,41,47]
NDR1/2	MST1/2	Thr444/Thr442	Crucial for kinase activity [38,41,43,44]
NDR1/2	MST3	Thr442	Crucial for kinase activity [45,46]
LATS1	Unknown mechanism#	Ser909	Essential for kinase activity [48,49]#
LATS1	MST1/2#	Thr1079	Essential for kinase activity [48,49]#
LATS1	Cdk1/cyclin B	Thr490	Might play a role in mitosis [50]
		Ser613	
LATS1	NUAK1	Ser464	Controls LATS1 protein stability [51]
LATS2	Aurora A	Ser83/Ser380	plays a role in mitosis [52-54]
LATS2	CHK1/2	Ser408	Plays a role in DNA damage signalling [55]
LATS2	PKA	Ser172	Regulate LATS2 activity towards YAP [56]
		Ser380	
		Ser592	
		Ser598	

#The potential auto- and trans-phosphorylation events on LATS1/2 (on Ser909/Ser872 and Thr1079/Thr1041) are not yet dissected. Therefore, we can only predict here that MST1/2 mainly phosphorylate Thr1079/Thr1041 on LATS1/2, based on the reported conserved regulatory mechanism between MST1/2 and NDR1/2 in human cells [43,44] and Hippo and Warts/Trc in fly cells [57].

Importantly, these regulatory mechanisms are more complicated in mammalian cells. MST1/2 kinases also phosphorylate hMOB1A/B on Thr12 and Thr35, thereby increasing the affinity of hMOB1A/B towards NDR1/2 [58]. Moreover, NDR1 deficient in hMOB1A/B binding cannot be phosphorylated by MST1 on Thr444 in S-phase arrested cells [43], suggesting that hMOB1A/B binding to NDR1/2 is required for the phosphorylation of both regulatory sites in human cells [39]. NDR1/2 are also regulated by binding to hMOB2, which is a level of regulation that does not exist with LATS1/2, since hMOB2 does not bind to LATS1/2 [39]. hMOB2 competes with hMOB1A/B for binding to the NTR of NDR1/2, where hMOB2 binding appears to be inhibitory, while hMOB1A/B binding is activating [59]. In addition, MICAL-1 (molecules interacting with CasL 1) competes with MST1 for binding to the HM of NDR1/2, thereby antagonizing MST1-induced NDR activation [60]. The TORC2 (target of rapamycin complex 2) can also interact with NDR1 in HeLa cells [61], however, whether this interaction influences NDR1/2 activity is yet to be determined. The subcellular localisation of NDR1/2 seems to provide a further level of regulation, since membrane targeting of NDR1/2 is sufficient to trigger NDR1/2 phosphorylation and activation [62].

LATS1/2 also contain the two conserved key regulatory phosphorylation sites of AGC kinases, the Ser909/872 AS and Thr1079/Thr1041 HM, respectively [4]. Both sites are phosphorylated in cells and are essential for LATS1 kinase activity, since LATS1 S909A or T1079A mutants are inactive [48,49]. Like with NDR1/2, LATS1 activity is counteracted by PP2A-mediated dephosphorylation of the AS and HM, since OA treatment of mammalian cells results in dramatically increased AS/HM phosphorylation and kinase activity of LATS1 [49,58]. hMOB1A/B also bind to the conserved NTR domain of LATS1/2 [49,63,64], but whether this affects the auto-phosphorylation activity of LATS1/2 is still undefined. Moreover, LATS1 deficient in hMOB1A/B binding is inactive and does not phosphorylate YAP [49,56], and in MOB1A/B double knock out (DKO) keratinocytes LATS1/2 phosphorylation on the HM is decreased, while MST1/2 appears unaffected [7], indicating that LATS1/hMOB1 complex formation is required for LATS1 phosphorylation and kinase activity. Additionally, hMOB1A/B protein stability is regulated by the Praja2 E3 ligase, which can influence LATS1/2 functionality by decreasing hMOB1A/B levels [65]. Phosphorylation of hMOB1A/B on Thr12 and Thr35 by MST1/2 further plays a role in regulating LATS1/hMOB1 complex formation, since T12A and T35A mutants of hMOB1A/B did not bind to LATS1 [58]. MST1/2 also phosphorylate LATS1/2 [48], but whether MST1/2 phosphorylate only the HM of LATS1/2 is currently unknown. However, in MST1/2-deficient liver cells, LATS1 S909 and T1079 phosphorylation was not impaired, although MOB1A/B phosphorylation on Thr12/35 was absent [6]. In mouse thymocytes MST1/2 phosphorylation of MOB1A/B also functions independent of LATS1/2 [66], strongly suggesting that LATS/MOB1 complex formation is not always essential for LATS phosphorylation/activation. The subcellular localisation of LATS1 is also likely to play a role in the regulation of LATS1 activity, since membrane targeting of LATS1 increased LATS1 activity [49]. Significantly, membrane targeting of Warts together with Mats was sufficient to reduce tissue growth in *Drosophila*[67], suggesting that the membrane bound pool of LATS1/2 might be physiologically relevant as well. In contrast, in mouse keratinocytes LATS1/2 seem to be activated by MST1/2 in the nucleus [68]. In summary, current evidence suggests that LATS1/2 are likely to be regulated in a similar fashion as already reported for NDR1/2 [4]. AS and HM phosphorylations of LATS1/2 are essential for kinase activation, but whether these specific phosphorylation events are regulated through hMOB1A/B-mediated auto-phosphorylation on Ser909/872 on one hand, and by MST1/2 phosphorylation of Thr1079/1041 on the other hand, is yet to be defined experimentally. Certainly, the subcellular localisation of LATS1/2 plays a role in their activation as well. Very likely, different subcellular pools of LATS1/2 are regulated differently dependent on the cell type specific context.

Besides these regulatory AS and HM phosphorylations, LATS1/2 kinases are controlled by additional phosphorylation events (Table 1). LATS1 is phosphorylated on Thr490/Ser613 by Cdk1/cyclin B, which could play a role in mitosis [50]. LATS2 is phosphorylated on Ser83/380 and Ser408 by Aurora A and CHK1/2, respectively, which seems to play a role in mitotic progression and DNA damage signalling [52-55]. Phosphorylation of LATS2 by PKA on Ser172/380/592/598 further stimulates LATS2 activity towards YAP [56]. Loss of PKA phosphorylation on LATS2 neither affects the LATS2/hMOB1 interaction, nor alters the AS and HM phosphorylations of LATS2, although the LATS2 kinase activity towards YAP is impaired [56]. This suggests that other activating factors such as changes in subcellular localisation and structural conformation might be dependent on PKA phosphorylation of LATS1/2. Moreover, phosphorylation of LATS1 by NUAK-1 (novel (nua) kinase family 1) on Ser464 controls LATS1 protein stability [51]. However, whether this phosphorylation event regulates the ubiquitin-mediated degradation of LATS1 by the Itch and WWP1 E3 ligases [69-71] is currently unknown. Furthermore, LATS1/2 protein stability can be controlled by HSP90 [72] and ROS-PKC delta signalling [73]. Significantly, LATS2 expression levels are further regulated on the transcriptional level by the transcription factors FOXP3 and p53 [74,75]. LATS2 expression is also regulated by TTP (tristetraprolin), an ARE (AU-rich element) binding protein that promotes the degradation of ARE-containing transcripts [76], and at least six different microRNAs have been shown to negatively regulate LATS2 expression [77-81].

Besides the regulatory protein-protein interaction (PPI) between hMOB1A/B and LATS1/2 kinases [39], mammalian Hippo signalling is regulated by additional PPIs which directly or indirectly affect LATS/NDR activity [2,82]. Due to the emphasis of this review, we focus on discussing reported direct PPIs of LATS1/2 with scaffolding/adaptor proteins. Scribble has been shown to link MST/LATS/YAP/TAZ complexes, thereby facilitating LATS phosphorylation of YAP/TAZ [83]. The Angiomotin proteins AMOT, AMOTL1 and AMOTL2 can bind to and activate LATS1/2 kinases [84], in addition to forming AMOT/YAP and AMOT/TAZ complexes [85-87]. The mammalian Ajuba LIM proteins, Ajuba, LIMD1 and WTIP, can interact with LATS1/2, which seems to decrease LATS1/2 phosphorylation of YAP [88,89], suggesting that Ajuba LIM proteins are negative regulators of LATS1/2 activity. Kibra overexpression appears to stimulate Thr1079 phosphorylation by binding to LATS1 [90], while depletion of Kibra caused a decrease of LATS1 phosphorylation on Ser909 [91]. However, the involvement of endogenous MST1/2 and hMOB1A/B in these regulatory PPIs are yet unknown, therefore it is currently not possible to describe in more detail how these scaffolding/adaptor proteins regulate LATS1/2.

### Substrates of mammalian LATS/NDR kinases

As already mentioned, LATS1/2 phosphorylation of YAP/TAZ is a key event of the canonical Hippo pathway (Tables 2 and 3). LATS1/2 phosphorylate YAP on Ser61/109/127/164/381 [29,92] and TAZ on Ser66/89/117/311 [93], which led to the definition of a HXRXXS/T consensus motif for LATS1/2 kinases. YAP phosphorylation on Ser127 increases 14-3-3 binding to YAP, which results in the cytoplasmic retention of inactive Ser127-phosphorylated YAP [9,29,94]. In contrast, phosphorylation of YAP on Ser381 regulates YAP protein stability [95]. TAZ phosphorylation on Ser89 and Ser311 follows a very similar principle, with Ser89 phosphorylation facilitating 14-3-3 binding and cytoplasmic retention of TAZ [93,96,97] and Ser311 phosphorylation regulating TAZ protein stability [98]. In summary, LATS1/2-mediated phosphorylation of YAP/TAZ trigger the inhibition of YAP/TAZ on at least two levels, namely (1) cytoplasmic retention/nuclear exclusion of YAP/TAZ, and (2) decreasing protein stability of YAP/TAZ [3].

**Table 2:** Summary of reported targeting motifs of LATS/NDR substrates

**Targeting motif**	**Kinase(s)**	**Target site**
HVRGD**pS**	LATS1/2	YAP on Ser61 [29,92]
HSRQA**pS**	LATS1/2	YAP on Ser109 [29,92]
HVRAH**pS**	LATS1/2	YAP on Ser127 [29,92]
HLRQS**pS**	LATS1/2	YAP on Ser164 [29,92]
HSRDE**pS**	LATS1/2	YAP on Ser381# [29,92]
HSRQS**pS**	LATS1/2	TAZ on Ser66 [93]
HVRSH**pS**	LATS1/2	TAZ on Ser89 [93]
HLRQQ**pS**	LATS1/2	TAZ on Ser117 [93]
HSREQ**pS**	LATS1/2	TAZ on Ser311 [93]
LRKTG**pS**	LATS1	MYPT1 on Ser445 [99]
GARRS**pS**	LATS2	14-3-3γ on Ser59 [55]
HVRTH**pT**	LATS2	Snail1 on Thr203 [100]
KRRQT**pS**	NDR1/2	p21 on Ser146 [45]
HRRIL**pS**	NDR1/2	AAK1 on Ser635 [101]
HTRNK**pS**	NDR/2	Rabin8(mouse) on Ser240 [101]
HTRNK**pS**	NDR2	Rabin8(human) on Ser272 [102]
**H**X**R**XX**pS/T**		

#S381 of YAP2 corresponds to S397 of YAP1.

Note:

PI4KB, Panx2, and Rab11fip5 sequences are not shown, since they are not yet confirmed as direct substrates of NDR1/2 [101]. However, these three substrates also display the HXRXXS/T motif [101].

**Table 3:** Summary of known direct downstream events/substrates of LATS/NDR kinases

**Kinase(s)**	**Substrate**	**Role of phosphorylation**
LATS1/2	YAP on Ser61	Not specifically determined [29,92]
LATS1/2	YAP on Ser109	Not specifically determined [29,92]
LATS1/2	YAP on Ser127	Facilitates 14-3-3 binding/cytoplasmic retention [9,29,94]
LATS1/2	YAP on Ser164	Not specifically determined [29,92]
LATS1/2	YAP on Ser381	Regulation of YAP protein stability [95]
LATS1/2	TAZ on Ser66	Not specifically determined [93]
LATS1/2	TAZ on Ser89	Facilitates 14-3-3 binding/cytoplasmic retention [93,96,97]
LATS1/2	TAZ on Ser117	Not specifically determined [93]
LATS1/2	TAZ on Ser311	Regulation of TAZ protein stability [98]
LATS1	MYPT1 on Ser445	Promotes MYPT1 phosphatase activity [99]
LATS2	14-3-3γ on Ser59	Regulation of 14-3-3γ in P-body formation [55]
LATS2	Snail on Thr203	Regulating of Snail1 protein stability [100]
NDR1/2	p21/Cip1 on Ser146	Regulates p21 protein stability [45]
NDR1/2	AAK1 on Ser635	Dendrite and spine development in neurons [101]
NDR1/2	Rabin8 on Ser240#	Dendrite and spine development in neurons [101]
NDR2	Rabin8 on Ser272#	Primary cilia biology [102]
NDR1/2	PI4KB on Ser277*	Not determined [101]
NDR1/2	Panx2 on Ser514*	Not determined [101]
NDR1/2	Rab11fip5 on Ser307*	Not determined [101]

#NDR kinases can phosphorylate the same conserved motif in mouse and human Rabin8.

*Potential substrates that have yet to be confirmed as direct substrates.

Not surprisingly, LATS1/2 have additional substrates in mammalian cells (Tables 2 and 3). LATS1 phosphorylates MYPT1 (myosin phosphatase-targeting subunit 1) on Ser445, thereby promoting MYPT1 phosphatase activity [99]. LATS2 also phosphorylates 14-3-3γ on Ser59 [55] and Snail1 on Thr203 [100], which influences P-body formation and Snail1 protein stability, respectively. The phosphorylation of 14-3-3 by LATS2 is particularly intriguing, since this might represent an additional regulatory level of the 14-3-3/YAP interaction that drives the cytoplasmic retention of inactive YAP. LATS2 can also phosphorylate DYRK1A (dual-specificity tyrosine-(Y)-phosphorylation-regulated kinase 1A), which enhances DYRK1A kinase activity and thereby possibly plays a role in RB-mediated senescence [103]. Significantly, the phosphorylation motifs in MYPT1 and 14-3-3γ do not align with the postulated HXRXXS/T consensus motif for LATS1/2 kinases, but rather display the basic R/KXXS/T motif, which is very common amongst AGC kinases [13].

NDR1/2 kinases have three documented substrates (Tables 2 and 3). NDR1/2 phosphorylate the cell cycle regulator p21/Cip1 on Ser146, thereby regulating p21 protein stability [45]. NDR1/2 also phosphorylate murine AAK1 (AP-2 associated kinase-1) and Rabin8 (Rab8 guanine nucleotide exchange factor) on Ser635 and Ser240, respectively [101]. NDR2 has further been shown to phosphorylate Rabin8 on Ser272 in human cells [102], which is the same site as previously reported for mouse Rabin8 phosphorylation on Ser240 (Tables 2 and 3). NDR1 can also phosphorylate YAP *in vitro*[92], however, the *in vivo* phosphorylation of YAP by NDR1/2 has not been documented so far. Intriguingly, two of three NDR1/2 substrates are also phosphorylated on the HXRXXS/T motif (Table 2), suggesting that the HXRXXS/T motif might be a common feature of LATS1/2 and NDR1/2 kinases. This speculation is further support by the notion that LATS1 and NDR1 display the same peptide substrate preferences *in vitro*, with the ideal substrate peptide containing the HXRXXS/T motif [92].

Of course, the substrate phosphorylations by LATS/NDR are counteracted by protein phosphatases to enable cells to rapidly adapt their signalling outputs, hence it is not surprising that PP1A was reported to mediate the dephosphorylation of YAP/TAZ [104,105]. PTPN14 (non-receptor tyrosine phosphatase 14) has also been shown to regulate YAP function, but whether this regulation is dependent of PTPN14 tyrosine phosphatase activity is currently debatable [106]. Possibly, PTPN14 influences the Tyr phosphorylation of YAP by c-Abl [107] than playing a role in counter balancing LATS1/2 substrate phosphorylation. However, since PKL01, a LATS/NDR homologue in plants, has recently been shown to be a dual-specificity kinase that can phosphorylate Ser/Thr and Tyr residues [108], it is tempting to speculate that it is just a question of time until the LATS/NDR substrate spectrum will be expanded to Tyr phosphorylations. Last, but not least, we would like to stress that current genetic evidence from *Drosophila* studies [5] suggests Warts (the fly counterpart of LATS1/2) is very likely to have additional substrates besides Yorkie (the fly counterpart of YAP/TAZ). In this context, it is noteworthy that Thompson and colleagues recently reported that Warts phosphorylates and inhibits the actin regulator Enabled, thereby restricting F-actin polymerization to local migrating clusters [109]. These findings suggest that the mammalian counterpart(s) of Enabled are very likely to also represent novel LATS1/2 substrates, besides pointing out that *Drosophila* genetics combined with biochemical approaches are likely to keep on pointing the way with regard to discovering novel LATS/NDR substrates.

### Functions of mammalian LATS/NDR kinases

In *Drosophila*, loss of the tumour suppressor Warts (the fly counterpart of mammalian LATS1/2) is larval lethal [16,17]. In contrast, LATS1 knock-out (KO) mice are viable [8], while LATS2 knock-out mice die during embryonic development, most likely due to defective cytokinesis resulting in genomic instability [64,110]. However, LATS1 null animals develop tumours [8], and immortalised LATS2 null MEFs display loss of contact inhibition [64,110], indicating that LATS1/2 might function as tumour suppressors in mammals [10]. LATS1/2 whole body DKO animals have not been reported yet. Nevertheless, a study combining LATS2 KO with RNAi depletion of LATS1 has shown that LATS1/2 kinases are crucial for early embryonic development [111]. More specifically, LATS1/2 are required to distinguish between trophectoderm and inner cell mass in preimplantation mouse embryos [111]. LATS1/2 also play a role in heart development [112,113], since interference with LATS1/2 function by either overexpressing dominant-negative LATS2 [112] or heart specific deletion of LATS1/2 [113] resulted in heart abnormalities. Taken together, LATS1/2 play important roles in embryonic development and heart formation, besides functioning as tumour suppressors in mammals.

While studies of tissue specific ablation of LATS1/2 function in animals are still limited in number, the roles of LATS1/2 as major regulators of the YAP/TAZ proto-proteins are well established [1,2]. In canonical Hippo signalling MST1/2 activated LATS1/2 phosphorylate YAP/TAZ on Ser127/89 and Ser381/311, respectively, thereby controlling YAP/TAZ on two levels: (1) Ser127/89-mediated spatial regulation (nuclear-cytoplasmic shuttling) and (2) Ser381/311-mediated phospho-degron mediated temporal regulation (degradation) (see Tables 2 and 3). However, the spatial regulation of YAP by LATS1/2 appears to be more complicated, since in sparse human and murine cell lines Ser127/112-phosphorylated YAP can also be detected in nuclei [114]. Moreover, the model of MST1/2-LATS1/2 mediated regulation of YAP has been challenged by studies of MST1/2 and YAP KO animals. First, liver specific deletion of MST1/2 in mice causes hepatocellular carcinoma (HCC) by YAP deregulation without any apparent involvement of LATS1/2 [6]. Second, YAP is negatively regulated in keratinocytes without any apparent involvement of MST1/2 or LATS1/2 signalling [115]. Third, in mouse thymocytes MST1/2 mainly signals through phosphorylation of MOB1A/B without any apparent involvement of LATS1/2 or YAP [66]. Fourth, in the mouse intestine YAP displays a growth-suppressive function restricting Wnt signals during intestinal regeneration [116]. These studies suggest that (1) MST1/2 does not always signal through LATS1/2 to YAP, (2) MST/LATS signalling are dispensable for YAP regulation in specific cell types, (3) MST1/2 signalling can function completely independent of the LATS/YAP signalling branch in specialised cell types, and (4) YAP does not always function as a proto-oncoprotein, but could also have context dependent tumour suppressive activity in the colon.

This last point is also supported by studies of breast cancer patients, which currently suggest that YAP might have oncogenic and tumour suppressive functions dependent on the breast cancer subtype [117]. Now a similar picture appears to evolve with respect to the role of YAP in colon cancer, since Camargo and colleagues found that YAP is silenced in a subset of highly aggressive and undifferentiated human colorectal carcinomas [118], while other studies suggest that YAP functions as a proto-oncoprotein in the colon [3]. The regulation of YAP in HCC is also not completely clear. In a significant fraction of human HCC samples Zhou et al. detected a correlation between decreased phospho-S127 YAP and decreased MST1 activity, arguing that MST1/2 activity is a key determinant upstream of YAP [6]. In contrast, Li et al. reported that in their HCC samples phospho-Ser127 YAP is decreased together with phosphorylated LATS1/2, while MST1/2 activity, as judged by the phosphorylation status of MST1/2, was not affected [119]. In summary, these findings illustrate that in human breast, colon and hepatic malignancies the role of the MST1/2-LATS1/2-YAP axis will most likely need to be defined based on cancer subtypes.

Significantly, LATS1/2 signalling could play a further role in mammalian stem cells [116,120]. YAP needs to be inactivated during the differentiation process of murine embryonic stem (ES) cells and elevated during iPS (induced pluripotent stem cells) reprogramming, illustrating that YAP is a critical regulator of stem cell pluripotency [121]. However, in these settings the regulation of YAP by LATS1/2 is yet to be defined. This point is important, since Zhou et al. showed that MST1/2 signalling plays a crucial role in YAP regulation in colon stem cells without apparent involvement of LATS1/2 signalling [122], suggesting that the regulation of YAP in stem cells might not always depend on LATS1/2. Nevertheless, knockdown of LATS2 can increase the efficiency of the formation of human iPS, most likely by releasing the normal repressive function of LATS2 as TAZ antagonist [79]. Therefore, LATS1/2 appear to be key players in mammalian stem cell biology, although much work is yet to be done to understand precisely how LATS1/2 function upstream of YAP/TAZ in this specific cell type.

Whatever the case might be in cancer and stem cells, in mostly normal human cell lines (in particular HEK293 and MCF10A cells) LATS1/2 function downstream of G-protein-coupled receptors (GPCRs) as central controllers of YAP/TAZ activities [2]. The Guan and Wu laboratories discovered that LPA (lysophosphatidic acid) and S1P (Sphingosine-1-phosphate) are major serum components responsible for YAP/TAZ activation [123,124]. More specifically, Yu et al. showed that LPA and S1P act through G12/13-coupled receptors to inhibit LATS1 S909/T1079 phosphorylation and activity, thereby allowing the dephosphorylation and consequent activation of YAP/TAZ [124]. Stimulation of protease-activated receptors (PARs; another group of GPCRs) also results in the inhibition of LATS1 activity due to decreased Ser909 and Thr1079 phosphorylation, which results in decreased YAP1 S127 phosphorylation, consequently allowing nuclear accumulation of active YAP [125]. Moreover, Guan and colleagues found that glucagon and epinephrine act through Gs-coupled receptors to stimulate LATS1 S909/T1079 phosphorylation, followed by phosphorylation and inhibition of YAP by activated LATS1/2 [124]. Significantly, these studies further suggest that GPCR signalling acts through Rho GTPases to trigger changes in LATS1/2 phosphorylation completely independent of MST1/2 signalling [124,125]. This raises the question how Ser909 and Thr1079 phosphorylation of LATS1/2 is regulated in this setting (see also Table 1). Potentially, changes in actin dynamics modulate these phosphorylation events [2], but the molecular mechanisms are currently not understood.

EGF (epidermal growth factor) signalling seems also to be able to regulate Hippo signalling in MCF10A cells [126], which is supported by a recent genetic study in *Drosophila*[89]. However, the role of EGF/EGFR signalling upstream of the Hippo pathway is currently debatable, since data from the Guan laboratory suggest that EGF has no significant effect on YAP phosphorylation [29,124]. In spite of these conflicting results, it is currently undisputed that LATS1/2 also function outside of the canonical Hippo pathway [127]. The Kolch and O’Neill laboratories have shown that K-ras signalling can function upstream of MST2-LATS1 in non-canonical Hippo signalling [128,129]. MST2 and LATS1/2 further play a role in Raf-1 activation by regulating the levels of the catalytic phosphatase subunit PP2A-C [130]. Moreover, LATS2 can regulate the levels of the p53 tumour suppressor by binding to Mdm2, the E3 ligase of p53 [75]. LATS1/2 have also been reported as regulators of different G1/S, G2/M, and mitotic cell cycle checkpoints, which have already been summarised elsewhere [14,127]. Taken together, LATS1/2 are central players in the regulation of YAP/TAZ functions in cancer and stem cell biology, although LATS1/2 also play significant roles in non-canonical Hippo signalling and even Hippo independent pathways.

While the activation mechanism of NDR1/2 is much better understood than the one of LATS1/2, much less is known about the biological functions of NDR1/2. NDR1 KO mice are viable, but develop T-cell lymphoma, most likely due to defective pro-apoptotic signalling [131]. NDR2 KO mice or NDR1/2 DKO animals have not been reported yet, however, dogs carrying a mutation in NDR2 display retinal degeneration [132]. Furthermore, it has been reported that human NDR1/2 play a role in centrosome duplication in S-phase [43,133], contribute to mitotic progression [134,135], and regulate the G1/S cell cycle transition by phosphorylating p21 [45]. Moreover, NDR1 regulates the protein stability of the proto-oncoprotein c-myc [45,136-138]. However, the mechanism of c-myc regulation by NDR1 is currently debatable, since Califano and colleagues reported that it is kinase activity dependent [136], while the Hemmings laboratory argues it is independent of NDR1 activity [45,137]. Taken together, these reports suggest that NDR1/2 are important cell cycle regulators. The regulation of the G1/S cell cycle transition by NDR1/2 can be explained by the negative regulation of the p21 cell cycle inhibitor combined with the positive regulation of c-myc levels [137]. However, the substrates functioning downstream of NDR1/2 in S-phase and mitosis are yet to be defined.

NDR1 functions additionally in apoptotic signalling [44,60,131], and has also been reported to play some role in oxidative stress MAPK (mitogen-activated protein kinase) signalling [139,140]. Furthermore, NDR2 has recently been described as regulator of ciliogenesis via phosphorylating Rabin 8 [102]. Last, but not least, Jan and colleagues reported recently [101] the identification of the first NDR1/2 substrates in neurons (see Tables 2 and 3). The authors functionally validated two substrates, showing that AAK1 and Rabin 8 function downstream of NDR1/2 in neuronal dendrite and synapse formation [101]. In summary, NDR1/2 function in the regulation of cell cycle progression, centrosome biology, stress/apoptotic signalling, and neuronal dendrite/synapse formation.

### Mammalian LATS/NDR kinases, centrosomes and the actin cytoskeleton

As already mentioned NDR1/2 kinases play a part in centrosome biology, most likely by associating with centrosomes [43,102,133]. LATS1/2 have also been detected on centrosomes [4], but the centrosomal function of LATS1/2 is currently not well understood. Nevertheless, two factors involved in centrosome-based ciliogenesis, NPHP4 and 9 (nephrocystin proteins 4 and 9), have been shown to regulate YAP/TAZ function [141,142]. NPHP4 interacts with LATS1 and inhibits LATS1 mediated phosphorylation of YAP and TAZ [141,142], while NPHP9 targets TAZ to the nucleus in a TAZ/NPHP9 complex [142]. Whether these regulatory processes involve cytoskeletal changes is currently unclear, although YAP/TAZ are downstream effectors of changes in the extracellular matrix, cell adhesion, cell shape and the cytoskeleton [143].

In particular, the actin cytoskeleton has recently gained more attention in the Hippo community. In *Drosophila* and human cells F-actin remodelling alters Hippo signalling [144]. Piccolo and colleagues found that YAP/TAZ are downstream of mechanical signals that are influenced by extracellular matrix rigidity and cell shape [145]. Significantly, this process is dependent on Rho GTPase activity and F-actin dynamics, but appears to be independent of LATS1/2 signalling [145]. The Sasaki laboratory also reported a regulation of YAP by cell morphology in an F-actin dependent manner, although their data suggest that LATS1/2 are involved in this process [114]. In support of the model which places F-actin dynamics upstream of LATS1/2 to regulate YAP, Zhao et al. showed that cell attachment and cytoskeleton remodelling regulates LATS1/2 activity and consequently YAP activity [146]. Moreover, in human cells GPCR signalling acts through F-actin remodelling to trigger changes in LATS1/2 activity towards YAP/TAZ [124,125]. Based on these findings the role of LATS1/2 in these settings is debatable, however, all these studies fully agree that YAP/TAZ function as sensors and mediators of mechanical inputs which are influenced by the cellular architecture and microenvironment.

Intriguingly, it has been reported that LATS1 can bind to actin and inhibit actin polymerisation [147]. Moreover, LATS1 interacts with Zyxin [148] and LIMK1 [149], two regulators of actin filament assembly. These findings suggest that LATS1 might also function in mechanosensing, maybe even independent of YAP/TAZ. Whatever the case, in *Drosophila*, mutation of Warts results in altered F-actin levels [150], suggesting that Warts is required for normal actin dynamics. Not surprisingly, the same study also showed that Trc mutants have altered levels of F-actin [150], because it has already been speculated since the year 2000 that the actin cytoskeleton might be a Trc target [15]. However, it is currently not established whether NDR1/2 kinases can also regulate F-actin remodelling, although a NDR2/actin complex has been reported nearly a decade ago [151]. In summary, actinomyosin dynamics play an important role in the control of the Hippo pathway.

## Conclusions

While the involvement of LATS/NDR in the regulation of the actin cytoskeleton is yet to be elucidated in more detail, it is undisputed that YAP/TAZ function as sensors and mediators of mechanical inputs coming from the cellular architecture and microenvironment. Besides F-actin remodelling, changes in the microtubule cytoskeleton should also to be considered in future studies, since the Guan laboratory could already show that LATS1/2 activity is modulated by anti-microtubule drugs [146]. It is noteworthy that hMOB1A/B, a key regulator of LATS/NDR kinases [39], has recently been shown to control microtubule dynamics [152], suggesting that LATS/NDR might also function as regulators of the microtubule cytoskeleton. The role of MST1/2 in cytoskeletal signalling is also not fully understood. Importantly, in this context, cell type dependent roles must be considered, since MST1/2 is dispensable for LATS1/2 signalling in MEFs, but not in HeLa cells [146].

Future research is further needed to decipher how LATS1/2 are regulated by hMOB1A/B and MST1/2, since currently the mechanism of NDR1/2 regulation by phosphorylation must serve as the model for LATS1/2 regulation. NDR1/2 are mainly controlled (1) by binding of hMOB1A/B to the NTR of NDR1/2 triggering auto-phosphorylation of NDR1/2 on the AS, and (2) by phosphorylation of NDR1/2 by MST1/2/3 on the HM. Phosphorylation of LATS1/2 on the conserved AS and HM regulatory sites is also essential for LATS1/2 kinase activity, but the molecular regulatory mechanisms of these phosphorylation events are currently not understood. The regulation of LATS/NDR activities is even more complex in cells, since MST1/2-mediated phosphorylation of hMOB1A/B influences hMOB1/LATS and hMOB1/NDR complex formation. In addition, changes in subcellular localisation, additional phosphorylation events, and competition between activating and inhibitory factors for kinase binding influence LATS/NDR activities. Furthermore, MST1/2 signalling is dispensable for LATS/NDR phosphorylation in selected cell types and biological functions, indicating that additional upstream kinases of LATS/NDR need to be studied in the future. In this context, it is worth mentioning that McCollum and colleagues recently reported that the activities of the yeast LATS/NDR kinases Sid2 and Orb6 are cross-regulated by Sid2 phosphorylating Nak1, the upstream Hippo kinase of Orb6 in yeast [153]. This raises the interesting possibility that human LATS1/2 and/or NDR1/2 might function upstream of each other in specific settings. However, this form of cross-regulation has yet to be reported in mammals.

To date, the best characterised LATS1/2 function is the regulation of YAP/TAZ by phosphorylation, thereby playing a crucial role in mammalian cancer and stem cell biology. In canonical Hippo signalling LATS1/2 phosphorylate YAP/TAZ on Ser127/89 and Ser381/311, respectively, thereby controlling YAP/TAZ on two levels, namely Ser127/89-mediated spatial regulation and Ser381/311-mediated temporal regulation. However, LATS1/2 also function in non-canonical Hippo signalling and even in Hippo independent pathways, thereby playing roles in Ras/Raf-1 signalling, the regulation of p53, and cell cycle progression. In contrast to LATS1/2, NDR1/2 functions have only recently been reported, proposing that NDR1/2 function in the regulation of cell cycle progression, centrosome biology, stress/apoptotic signalling, and neuronal dendrite/synapse formation. The recently reported mitochondrial role of Trc [154] will potentially provide a further platform to discover more roles of NDR1/2 in mammals. Taken together, given the recent research progress on LATS/NDR functions, we believe that more key functions of LATS/NDR are yet to be discovered, in particular with respect to NDR1/2. More specifically, the putative roles of LATS/NDR as sensors and mediators of internal and external mechanical forces, upstream of YAP/TAZ, are exciting avenues to be explored in the future.

## Abbreviations

YAP: Yes-associated protein; TAZ: Transcriptional co-activator with PDZ-binding motif; MST: Mammalian Ste20-like serine/threonine kinase; MOB: mps one binder; Mats: mob as tumour suppressor; AGC: Protein kinase A (PKA)/PKG/PKC-like; LATS: Large tumour suppressor; NDR: Nuclear dbf2 related; STK38/STK38L: serine/threonine kinase 38/38L; Trc: Tricornered; NTR: N-terminal regulatory domain; AS: Activation segment motif; HM: Hydrophobic motif; MEN: Mitotic exit network; SIN: Septation initiation network; OA: Okadaic acid; PP2A: Protein phosphatase type 2A; KO: Knock-out; DKO: Double knock-out; PPI: Protein-protein interaction; MYPT1: Myosin phosphatase-targeting subunit 1; Rab8: Guanine nucleotide exchange factor (Rabin 8); AP-2: Associated kinase-1 (AAK1).

## Competing interests

The author declares that he has no competing interests.
